# The Impact of Outliers on Net-Benefit Regression Model in Cost-Effectiveness Analysis

**DOI:** 10.1371/journal.pone.0065930

**Published:** 2013-06-19

**Authors:** Yu-Wen Wen, Yi-Wen Tsai, David Bin-Chia Wu, Pei-Fen Chen

**Affiliations:** 1 Clinical Informatics and Medical Statistics Research Center, College of Medicine, Chang Gung University, Tao-Yuan, Taiwan; 2 Institute of Health and Welfare Policy, National Yang-Ming University, Taipei, Taiwan; 3 Discipline of Pharmacy, Monash University Sunway Campus, Kuala Lumpur, Malaysia; 4 Division of Environmental Health and Occupational Medicine, National Health Research Institutes, Miaoli, Taiwan; UNAIDS, Switzerland

## Abstract

Ordinary least square (OLS) in regression has been widely used to analyze patient-level data in cost-effectiveness analysis (CEA). However, the estimates, inference and decision making in the economic evaluation based on OLS estimation may be biased by the presence of outliers. Instead, robust estimation can remain unaffected and provide result which is resistant to outliers. The objective of this study is to explore the impact of outliers on net-benefit regression (NBR) in CEA using OLS and to propose a potential solution by using robust estimations, i.e. Huber *M*-estimation, Hampel *M*-estimation, Tukey's bisquare *M*-estimation, *MM*-estimation and least trimming square estimation. Simulations under different outlier-generating scenarios and an empirical example were used to obtain the regression estimates of NBR by OLS and five robust estimations. Empirical size and empirical power of both OLS and robust estimations were then compared in the context of hypothesis testing.

Simulations showed that the five robust approaches compared with OLS estimation led to lower empirical sizes and achieved higher empirical powers in testing cost-effectiveness. Using real example of antiplatelet therapy, the estimated incremental net-benefit by OLS estimation was lower than those by robust approaches because of outliers in cost data. Robust estimations demonstrated higher probability of cost-effectiveness compared to OLS estimation. The presence of outliers can bias the results of NBR and its interpretations. It is recommended that the use of robust estimation in NBR can be an appropriate method to avoid such biased decision making.

## Introduction

Regression technique has been widely used in cost-effectiveness analysis (CEA) to control confounding variables in modelling for patient-level data [1]–[4]. Ordinary least squares (OLS) estimation, which minimizes the sum of squares of error, is the most common approach used to find a best-line of predicted values because OLS estimation provides a best linear unbiased estimator (BLUE) among the class of linear ones [5]. However, OLS estimation can be affected by the presence of outliers, observations which deviate far from the linear relation of the response variable and the exploratory variables [6]. Though outliers usually bias the OLS predictions towards outliers, they are often embedded in empirical analysis.

In general, outliers can be roughly classified into two types: man-made one and random one [7]. Man-made outliers may be arising because of typographical error, mis-reporting information involving private matters such as salary and drug abuse, incorrect distribution assumption and sampling error; random outliers may be arising because of random chance for drawing sample from a population [8]. Presence of man-made or random outlier, or both, would seriously influence the results of statistical analyses including point and interval estimates, and type I and type II errors [8], [9]. Some man-made outliers can be avoided by a strict data entry and rechecking processes before conducting a statistical analysis. Data transformation is another way to reduce the influence of outliers. However it may be not appropriate for hypothesis testing and straightforward interpretation becomes difficult using transformed data [8]. Aside from data transformation, removing outliers from the database directly is a simple practice to avoid the problem. However, arbitrarily removing some data from a database may lead to sample selection bias which can be considered as a specification error in linear regression [10] and potentially threats internal validity [11]. In most cases, outliers are hard to identify particularly when data are multi-dimensional. In addition, some outliers are hard to detect because they are masked by other outliers. That is referred to a masking effect [12]. Instead of data transformation or removing data, robust methods can provide an alternative approach to deal with outliers without deleting them.

OLS estimator is extremely sensitive to multiple outliers in linear regression analysis. It can even be easily biased by just a single outlier because of its low breakdown point [6] which is defined as the percentage of outliers allowed in a dataset for an estimator to remain unaffected [13]. The breakdown point of OLS estimator equals to the inverse of the sample size which would tend to zero as the sample size tends to grow large [6]. Unlike OLS estimator, robust regression provides robust regression estimators even in the presence of multiple outliers. The impact of outliers when using robust regression is minimized by giving smaller weight for outliers in the estimation procedure [14]. So far, several robust regression estimators have been proposed. The simplest robust approach of robust regression is *M-estimation* and its variant is general *M-estimation* [15]–[17]. Least trimmed squares (LTS) estimation is a robust method with high breakdown point, which can withstand high proportion of outliers and still maintains its robustness [18]. *MM*-estimation has both high breakdown point and higher statistical efficiency [19].

In CEA studies, outliers are more frequently observed in cost data than effectiveness data. The conventional strategy to deal with outliers is to estimate incremental cost-effectiveness ratio (ICER) by including and excluding outliers in order to see how they impact ICER [20]–[24]. In those studies, the estimates of ICER when including outliers were larger than those when excluding them. Therefore, analyzing cost-effective data with and without outliers can lead to different CEA results. Furthermore, the proportions of outliers were reported to be less than 10% and this could be underestimated because of masking effect. Sometimes, the influence of outliers on ICER could only be minor when the proportion of outliers is relatively small, and they may then be excluded directly without much concern. However, it might be questionable to inform decision makers by simply presenting cost-effectiveness results by including and excluding outliers. Up to now, only one study has investigated how presence of outliers (3%, 5% and 10% of outliers assumed in the data) in cost data would impact the precision of confidence interval for ICER estimated by both bootstrapping method and Fieller's theorem [25]. The results showed that presence of outliers would affect the coverage probability of the confidence interval of ICER. However, impact of outliers on regression-based CEA and the way to tackle the problem have not been addressed.

The objective of this study is to evaluate the impact of outliers on a net-benefit regression (NBR), a kind of regression-based CEA, using a number of simulated scenarios where cost outliers were generated and a real dataset. The outliers were assumed to occur randomly in the cost variable and to be larger than usual values of the cost variable in the simulation. The different simulation scenarios were considered and described in the following section. An empirical example of antiplatelet therapy in the management of cardiovascular diseases was presented to demonstrate the impact on the probability and critical value of cost-effectiveness, especial on the cost-effectiveness acceptability curve (CEAC), which provides a summary for acceptability of cost-effectiveness with a range of willingness-to-pay (WTP) [26].

## Methods

Consider a cost-effectiveness study which compares two arms (Arm 1 vs. Arm 0), data for the effect (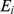), the cost (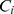) and the corresponding covariates (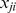), *j* = 1, 2,…, *s* of each subject *i*, *i* = 1, 2,…, *n*, were collected. Then, net-benefit value (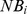) for each subject *i* can be expressed as
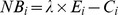
given a maximum acceptable WTP per unit of effectiveness, 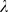.

### The NBR Framework

The relationship between 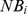 and 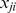 can be expressed as a linear regression:
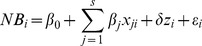
where 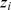 is an indicator variable (0 for Arm 0 and 1 for Arm 1), 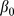, 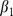,…, 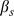 and 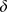 are regression parameters and 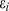 is the error term. Compared with Arm 0, the incremental net-benefit of Arm 1 is the estimated regression parameter 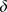 on the treatment indicator. This model is usually referred to a NBR [4]. In this model, Arm 1 is considered cost-effective if the incremental net-benefit, 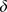, is positive and not cost-effective if 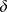 is non-positive. With regard to the sampling uncertainty, the following statistical hypothesis can be tested for cost-effectiveness of Arm 1:
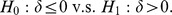



The computation of a p-value for this one-sided test and the point estimates and inferences for the NBR are well-documented and the CEAC can be plotted by varying 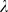 from 0 to a large value on the horizontal axis and the corresponding probabilities of cost-effectiveness on vertical axis. Therefore, the probability of cost-effectiveness is calculated as 1 minus the p-value of the above test [4], [27].

### Robust Estimations for the NBR

A large number of estimation approaches can provide robust estimates for a linear regression including *M*-estimator and its variants [15]–[17], [28], [29], least-median estimator [18], LTS estimator [18], *MM*-estimator [19], least-absolute estimators [30], *S*-estimator [31], two-stage estimator [32] and so on. Comparative studies of robust estimators and OLS estimator based Monte Carlo simulation or real examples have been published but the results in term of bias, efficiency, test of the null hypothesis and forecast ability of those estimators were inconsistent [9], [33]–[38]. *MM*-estimator was better than OLS estimator and the other robust estimator in relative efficiency, bias and the statistical test [9]; *M*-estimator and LTS estimator outperformed OLS estimator on predicted valued of the dependent variables [33], [34]; *M*-estimator performed better than LTS estimator and *MM*-estimator on R-square [35]; *MM*-estimator and LTS estimator provider a higher R-square that OLS estimator and the estimates from *MM*-estimator and LTS estimator were very closed [36]; Tukey's bisquare *M*-estimator was performed better on effect estimates than Huber *M*-estimation and OLS estimator for experimental design data [37]; robust estimates showed the better predicted ability [38]. The inconsistent results were possibly caused by the difference in data structures or simulation scenarios. Previous study suggested that the choice of robust estimation would depend on the structure of data and users' discretion [39]. In this study, five robust estimations (Huber *M*-estimation, Hampel *M*-estimation, Tukey's bisquare *M*-estimation, *MM*-estimation and LTS) were used to illustrate the impact of outliers on NBR in CEA study compared with OLS estimation. These five estimations were discussed extensively in those comparative studies and supported in the standard statistical packages.

For parsimony purpose, the NBR mentioned above can be re-expressed as follows:
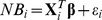
where 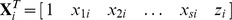 and the fitted model is
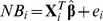
where 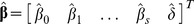 denotes the estimate of 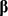 and 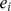 is the corresponding residual. This study uses one classical and five robust approaches to estimate the regression parameter 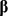: including ordinary least square estimation, three types of *M*-estimation [15]–[17], *MM*-estimation [19] and LTS estimation [18].

The most common robust estimation of a linear regression model is *M*-estimation [15]. The general *M*-estimator 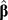 minimizes the following finite summation
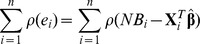
where 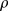 is a symmetric function which contributes to residual. In this paper, four types of the function 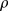 are included:
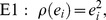


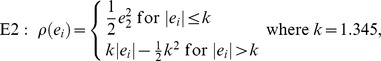


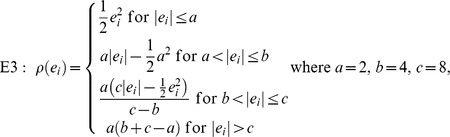


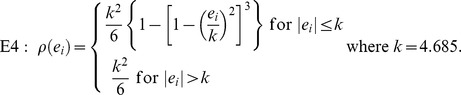



The function 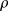 in E1 is for ordinary least squares estimation, E2 is for Huber *M*-estimation [15], E3 is for Hampel *M*-estimation [16], and E4 is for Tukey's bisquare *M*-estimation [17], respectively. *MM*-estimation was based on an *M*-estimator starting at the coefficients given by *S*-estimator and with fixed scale given by *S*-estimator [40]. Least trimming square (LTS) estimation is based on minimizing
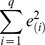
where 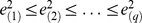 are the ordered squared residuals [18].

## Results

### Simulation Analysis

We designed a simulation study to illustrate the potential impact of outliers in CEA using NBR on determining cost-effectiveness of Arm 1 based on the comparison of six estimation procedures, i.e. OLS estimation, Huber *M*-estimation, Hampel *M*-estimation, Tukey's bisquare *M*-estimation, *MM*-estimation and LTS estimation as detailed in the following section.

#### Simulation Design

The effect (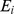) and cost (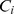) of the subject *i* is generated randomly from a bivariate normal distribution as
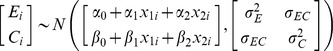
where 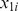 is a dummy regressor which is generated from a Bernoulli distribution with probability 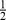, indicating that the subject belongs to Arm 0 (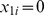) or Arm 1 (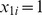) and 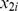 is a continuous regressor which is generated from a normal distribution with mean 2 and standard deviation 0.5. The parameters 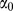, 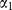 and 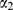 are all assumed to be 1; 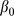 is assumed to be 50, 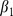 is assumed to be 10 and 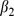 is assumed to be 1. So, compared with the subjects in Arm 0, the subjects in Arm 1 will benefit one unit of effect (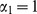) but cost 10 more dollars (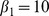). The covariance matrix is set to be
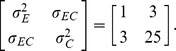



Those first 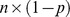 simulated samples were considered as regular cases (non-outliers), where 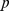 was the proportion for outliers in 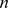 samples. For outlier sample, we assumed that the outliers just only occur in cost variable (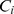) and last 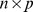 observations were denoted by outliers. Based on previous literatures and potential masking effect, the proportion of outlier 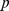 was set to be 0.05, 0.1, 0.2 and 0.3. Outlier samples were generated from three scenarios described as follows:

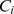
 was randomly drawn from a normal distribution with mean 150 and variance 1 for 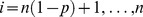.

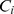
 was randomly drawn from a normal distribution with mean 200 and variance 1 for 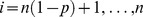.

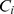
 was randomly drawn from a normal distribution with mean 150 and variance 1 for 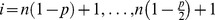 and drawn from a normal distribution with mean 200 and variance 1 for 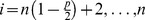.

#### Performance Comparison

For each set of parameter design, sample size (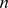 = 100, 500 and 1000) and WTP (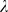 = 7, 8, 12 and 13), 500 independent data sets were created and six estimation procedures were applied to analyse each data set. After 500 repetitions, one quantity for each estimation procedure was calculated:
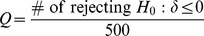
where *Q* was referred to the empirical size for 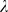 = 7 and 8 (i.e. 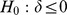 is true, but rejected) and the empirical power for 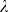 = 12 and 13 (i.e. 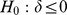 is false and rejected). The empirical size and empirical power were used to illustrate type I error and power (1-type II error) in 500 repetitions among different estimation procedures respectively.

#### Simulation Results

The results of the empirical size and empirical power were showed in Table 1 and Table 2, respectively. In Table 1, most empirical sizes were below a significance level saying 0.05 except for some cases in 20% and 30% of outliers. Table 2 showed that three *M*-estimations, *MM*-estimation and LTS estimation had higher empirical powers than OLS estimation. However, two robust procedures, Huber *M*-estimation and Hampel *M*-estimation, had lower empirical power than OLS estimation when the proportion of outliers achieved 30%. In the scenario of small sample size (*n* = 100), all empirical powers were less than 0.5 among all estimations; in contrast, in scenarios of large sample size (*n* = 500 or 1000), most empirical powers were over 0.5 except for OLS estimation. In short, as sample size increased, the empirical sizes decreased while empirical powers increased. Among the robust estimations, empirical powers of three *M*-estimations decreased dramatically as the proportion of outlier increased while the estimated powers of *MM*-estimation and LTS estimation slightly decreased. Larger WTP would lead to a smaller empirical size and larger empirical power.

**Table 1:** Empirical sizes for six estimation procedures in different combinations of simulation parameters.

			True cost for one unit of effectiveness = 10																							
Outlier Distribution			I. N(150,1)								II. N(200,1)								III. N(150,1) or N(200,1)							
Outlier Proportion			5%		10%		20%		30%		5%		10%		20%		30%		5%		10%		20%		30%	
WTP			7	8	7	8	7	8	7	8	7	8	7	8	7	8	7	8	7	8	7	8	7	8	7	8
Sample size	100	OLS estimation	0.02	0.02	0.02	0.04	0.04	0.06	0.07	0.08	0.02	0.02	0.02	0.03	0.05	0.06	0.07	0.08	0.00	0.01	0.03	0.03	0.05	0.05	0.05	0.06
		Huber *M*-estimation	0.00	0.00	0.00	0.00	0.00	0.01	0.07	0.07	0.00	0.00	0.00	0.00	0.00	0.01	0.07	0.07	0.00	0.00	0.00	0.00	0.00	0.00	0.03	0.04
		Hampel *M*-estimation	0.00	0.00	0.00	0.00	0.00	0.01	0.07	0.08	0.00	0.00	0.00	0.00	0.01	0.01	0.07	0.08	0.00	0.00	0.00	0.00	0.00	0.00	0.05	0.06
		Tukey's bisquare *M*-estimation	0.00	0.00	0.00	0.00	0.00	0.00	0.06	0.07	0.00	0.00	0.00	0.00	0.00	0.00	0.07	0.07	0.00	0.00	0.00	0.00	0.00	0.00	0.03	0.04
		*MM*-estimation	0.00	0.00	0.00	0.00	0.00	0.00	0.00	0.00	0.00	0.00	0.00	0.00	0.00	0.00	0.00	0.00	0.00	0.00	0.00	0.00	0.00	0.00	0.00	0.00
		LTS estimation	0.00	0.00	0.00	0.00	0.00	0.00	0.00	0.00	0.00	0.00	0.00	0.00	0.00	0.00	0.00	0.00	0.00	0.00	0.00	0.00	0.00	0.00	0.00	0.00
	500	OLS estimation	0.00	0.01	0.01	0.01	0.03	0.06	0.06	0.07	0.01	0.01	0.02	0.04	0.03	0.05	0.05	0.07	0.00	0.01	0.01	0.03	0.05	0.07	0.05	0.07
		Huber *M*-estimation	0.00	0.00	0.00	0.00	0.00	0.00	0.02	0.03	0.00	0.00	0.00	0.00	0.00	0.00	0.02	0.02	0.00	0.00	0.00	0.00	0.00	0.00	0.01	0.02
		Hampel *M*-estimation	0.00	0.00	0.00	0.00	0.00	0.00	0.06	0.07	0.00	0.00	0.00	0.00	0.00	0.00	0.05	0.07	0.00	0.00	0.00	0.00	0.00	0.00	0.05	0.07
		Tukey's bisquare *M*-estimation	0.00	0.00	0.00	0.00	0.00	0.00	0.02	0.04	0.00	0.00	0.00	0.00	0.00	0.00	0.02	0.04	0.00	0.00	0.00	0.00	0.00	0.00	0.00	0.00
		*MM*-estimation	0.00	0.00	0.00	0.00	0.00	0.00	0.00	0.00	0.00	0.00	0.00	0.00	0.00	0.00	0.00	0.00	0.00	0.00	0.00	0.00	0.00	0.00	0.00	0.00
		LTS estimation	0.00	0.00	0.00	0.00	0.00	0.00	0.00	0.00	0.00	0.00	0.00	0.00	0.00	0.00	0.00	0.00	0.00	0.00	0.00	0.00	0.00	0.00	0.00	0.00
	1000	OLS estimation	0.00	0.00	0.01	0.02	0.01	0.05	0.05	0.10	0.00	0.01	0.00	0.01	0.04	0.06	0.04	0.08	0.00	0.01	0.01	0.03	0.04	0.06	0.06	0.09
		Huber *M*-estimation	0.00	0.00	0.00	0.00	0.00	0.00	0.00	0.01	0.00	0.00	0.00	0.00	0.00	0.00	0.01	0.02	0.00	0.00	0.00	0.00	0.00	0.00	0.01	0.03
		Hampel *M*-estimation	0.00	0.00	0.00	0.00	0.00	0.00	0.05	0.10	0.00	0.00	0.00	0.00	0.00	0.00	0.04	0.08	0.00	0.00	0.00	0.00	0.00	0.00	0.05	0.08
		Tukey's bisquare *M*-estimation	0.00	0.00	0.00	0.00	0.00	0.00	0.01	0.02	0.00	0.00	0.00	0.00	0.00	0.00	0.02	0.02	0.00	0.00	0.00	0.00	0.00	0.00	0.00	0.00
		*MM*-estimation	0.00	0.00	0.00	0.00	0.00	0.00	0.00	0.00	0.00	0.00	0.00	0.00	0.00	0.00	0.00	0.00	0.00	0.00	0.00	0.00	0.00	0.00	0.00	0.00
		LTS estimation	0.00	0.00	0.00	0.00	0.00	0.00	0.00	0.00	0.00	0.00	0.00	0.00	0.00	0.00	0.00	0.00	0.00	0.00	0.00	0.00	0.00	0.00	0.00	0.00

**Table 2:** Empirical powers for six estimation procedures in different combinations of simulation parameters.

			True cost for one unit of effectiveness = 10																							
Outlier Distribution			I. N(150,1)								II. N(200,1)								III. N(150,1) or N(200,1)							
Outlier Proportion			5%		10%		20%		30%		5%		10%		20%		30%		5%		10%		20%		30%	
WTP			12	13	12	13	12	13	12	13	12	13	12	13	12	13	12	13	12	13	12	13	12	13	12	13
Sample size	100	OLS estimation	0.12	0.17	0.13	0.14	0.13	0.17	0.13	0.14	0.09	0.11	0.09	0.11	0.10	0.12	0.12	0.12	0.11	0.13	0.10	0.13	0.11	0.13	0.11	0.12
		Huber *M*-estimation	0.21	0.32	0.20	0.29	0.15	0.20	0.11	0.12	0.22	0.30	0.22	0.30	0.13	0.20	0.10	0.11	0.22	0.33	0.18	0.29	0.13	0.18	0.08	0.09
		Hampel *M*-estimation	0.22	0.34	0.21	0.28	0.13	0.15	0.13	0.14	0.24	0.37	0.25	0.38	0.23	0.33	0.12	0.12	0.24	0.37	0.22	0.33	0.17	0.22	0.10	0.12
		Tukey's bisquare *M*-estimation	0.22	0.34	0.24	0.34	0.21	0.31	0.11	0.12	0.23	0.34	0.27	0.37	0.21	0.35	0.11	0.11	0.26	0.39	0.23	0.35	0.23	0.32	0.14	0.17
		*MM*-estimation	0.22	0.34	0.23	0.35	0.20	0.30	0.15	0.16	0.22	0.34	0.26	0.37	0.21	0.35	0.21	0.30	0.26	0.39	0.23	0.35	0.22	0.32	0.14	0.18
		LTS estimation	0.27	0.39	0.27	0.40	0.22	0.32	0.23	0.31	0.28	0.40	0.28	0.41	0.23	0.34	0.20	0.30	0.31	0.43	0.24	0.37	0.24	0.35	0.19	0.26
	500	OLS estimation	0.34	0.49	0.25	0.38	0.32	0.43	0.36	0.43	0.22	0.36	0.21	0.29	0.18	0.25	0.22	0.26	0.27	0.41	0.22	0.31	0.22	0.28	0.25	0.32
		Huber *M*-estimation	0.65	0.86	0.51	0.78	0.36	0.55	0.19	0.27	0.65	0.86	0.56	0.80	0.35	0.54	0.08	0.12	0.63	0.87	0.52	0.79	0.35	0.53	0.15	0.21
		Hampel *M*-estimation	0.69	0.90	0.53	0.77	0.15	0.25	0.36	0.43	0.71	0.91	0.70	0.90	0.61	0.87	0.22	0.26	0.71	0.90	0.62	0.83	0.38	0.59	0.24	0.30
		Tukey's bisquare *M*-estimation	0.72	0.91	0.66	0.91	0.60	0.83	0.20	0.29	0.70	0.90	0.70	0.90	0.60	0.87	0.12	0.16	0.70	0.91	0.66	0.89	0.67	0.88	0.49	0.50
		*MM*-estimation	0.72	0.91	0.66	0.91	0.61	0.83	0.49	0.51	0.70	0.90	0.70	0.90	0.60	0.88	0.58	0.82	0.71	0.91	0.67	0.89	0.67	0.88	0.53	0.67
		LTS estimation	0.73	0.90	0.67	0.91	0.60	0.86	0.62	0.83	0.72	0.91	0.73	0.89	0.61	0.87	0.59	0.83	0.71	0.91	0.68	0.90	0.69	0.90	0.60	0.82
	1000	OLS estimation	0.51	0.78	0.50	0.70	0.48	0.63	0.58	0.71	0.34	0.49	0.27	0.41	0.30	0.37	0.32	0.40	0.40	0.64	0.39	0.53	0.33	0.46	0.44	0.54
		Huber *M*-estimation	0.88	0.99	0.82	0.96	0.56	0.76	0.27	0.39	0.89	0.99	0.81	0.98	0.56	0.81	0.10	0.15	0.87	0.99	0.82	0.97	0.57	0.78	0.20	0.32
		Hampel *M*-estimation	0.92	0.99	0.82	0.96	0.24	0.40	0.58	0.71	0.93	1.00	0.91	0.99	0.90	0.99	0.32	0.40	0.91	1.00	0.91	0.99	0.56	0.83	0.40	0.51
		Tukey's bisquare *M*-estimation	0.93	1.00	0.92	0.99	0.87	0.99	0.32	0.43	0.92	1.00	0.90	0.99	0.89	0.99	0.14	0.21	0.92	1.00	0.93	1.00	0.86	0.99	0.77	0.69
		*MM*-estimation	0.93	1.00	0.92	0.99	0.87	0.99	0.71	0.75	0.92	1.00	0.90	0.99	0.89	0.99	0.86	0.97	0.92	1.00	0.93	1.00	0.86	0.99	0.79	0.91
		LTS estimation	0.92	0.99	0.92	0.99	0.87	0.99	0.86	0.98	0.93	1.00	0.90	0.99	0.90	0.99	0.87	0.93	0.92	1.00	0.93	1.00	0.86	0.99	0.86	0.98

### Empirical Example: Antiplatelet Therapy

In this section, we used a real example of antiplatelet therapy, which provided prevention of cardiovascular diseases (CVD) to demonstrate different estimation scenarios of the NBR.

#### Background

Antiplatelet therapy which includes an administration of low-dose aspirin (75–150 mg) and clopidogrel, is effective as a secondary prevention for some CVD. Patients with aspirin treatment may have some level of gastrointestinal (GI) bleeding, and clopidogrel is aimed to reduce the occurrence of GI bleeding. A previous CEA has showed that aspirin plus proton-pump inhibitors (PPIs) was more cost-effective than clopidogrel with respect to hospitalization because of GI complications [41]. This study focused on those patients who had a medical history of GI bleeding and compared the cost-effectiveness with respect to outpatient visit between aspirin plus PPIs and clopidogrel. This study was conducted from Taiwanese healthcare payer perspective.

#### Data Source

The data were drawn from the Taiwan National Health Insurance Research Database (NHIRD) during year 2001 and 2006. Study subjects with one-year follow-up starting from the discharged date were classified into two groups, based on the antiplatelet therapy regimens they received during the 90 days following the hospital discharge due to major GI complications: (1) clopidogrel group: those who have been prescribed clopidogrel alone and (2) aspirin plus PPIs: those who have been prescribed aspirin plus PPIs.

#### Effect, Cost and Covariates

Effect variable was the number of days between the discharge date and the first time of outpatient service for GI illness including bleeding and perforation after discharge; the unit for the effect was days with maximum of 365 days. Cost variable was defined as the accumulated medical cost during the observation period (time to event for GI cases and 365 days for non-GI cases), including all medical expenses for inpatient and outpatient visits of CVD events and inpatient visits of GI events. The unit for cost variable was NTD (New Taiwan Dollars). Subject's age, gender, medicine use (DDD (define daily dose) for clopidogrel, aspirin plus PPIs), and medical history prior the follow-up (diabetes mellitus, cardiovascular diseases and lung-related diseases) were included as control variables in NBR.

#### Cos-effectiveness Analysis

A NBR analysis based on OLS estimation was initially used to compare the cost-effectiveness between the groups of aspirin plus PPIs and the clopidogrel group given a set of 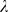 (the maximum acceptable WTP per unit of effectiveness). The results of preliminary analysis showed that there were some potential outliers in the cost variable. Because of the presence of those outliers, robust estimation procedures were then used to analyse the data. For each 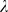 (50, 100, 150, and 200), the estimates on treatment effect, the corresponding one-sided p-values, and the probabilities of cost-effectiveness (calculated by both regression and bootstrapping) were summarized for comparison. The CEACs were also conducted for both OLS and robust estimation procedures.

#### Empirical Results

Table 3 showed the baseline characteristics of total sample of 649 subjects. Among them, 564 (87%) subjects used aspirin plus PPIs and 85 (13%) subjects used clopidogrel. In terms of the effects, aspirin plus PPIs group had longer delay on seeking outpatient care for GI illness than clopidogrel group (270.78 days/SD = 117.00 vs 250.84/SD = 121.75). Regarding the cost, the mean costs for aspirin plus PPIs were 27210 NTD (SD = 99648) and 22384 NTD (SD = 46918) for clopidogrel groups. Over 60% were males, and the mean age was about 72 years among the total sample. There were overall about 15% of study subjects who had medical history of diabetes mellitus, cardiovascular disease or lung-related diseases during one year prior to the entry into the study.

**Table 3:** Patients' baseline characteristics, medical history, and medication use during the follow-up.

	Total Sample (n = 649)	Aspirin+PPIs (n = 564)	Clopidogrel (n = 85)
Cost	26578±94420	27210±99648	22384±46918
Effectiveness	268.17±117.73	270.78±117.00	250.84±121.75
Age	72.08±10.69	71.98±10.77	72.71±10.16
Gender			
Female, n (%)	239 (37)	206 (37)	33 (39)
Male, n (%)	410 (63)	358 (63)	52 (61)
Medical history			
Diabetes mellitus, n (%)			
Yes	109 (17)	98 (17)	11 (13)
No	540 (63)	466 (83)	74 (87)
Cardiovascular disease, n (%)			
Yes	95 (15)	84 (15)	11 (13)
No	554 (85)	480 (85)	74 (87)
Lung-related diseases, n (%)			
Yes	107 (16)	94 (17)	13 (15)
No	542 (84)	470 (83)	72 (85)
Medication use during the follow-up			
Clopidogrel (DDDs)	23.69±78.79	-	180.89±138.26
Aspirin (DDDs)	112.45±126.13	129.39±126.94	-
PPIs (DDDs)	79.14±88.42	91.07±88.94	-

**Figure 1:**
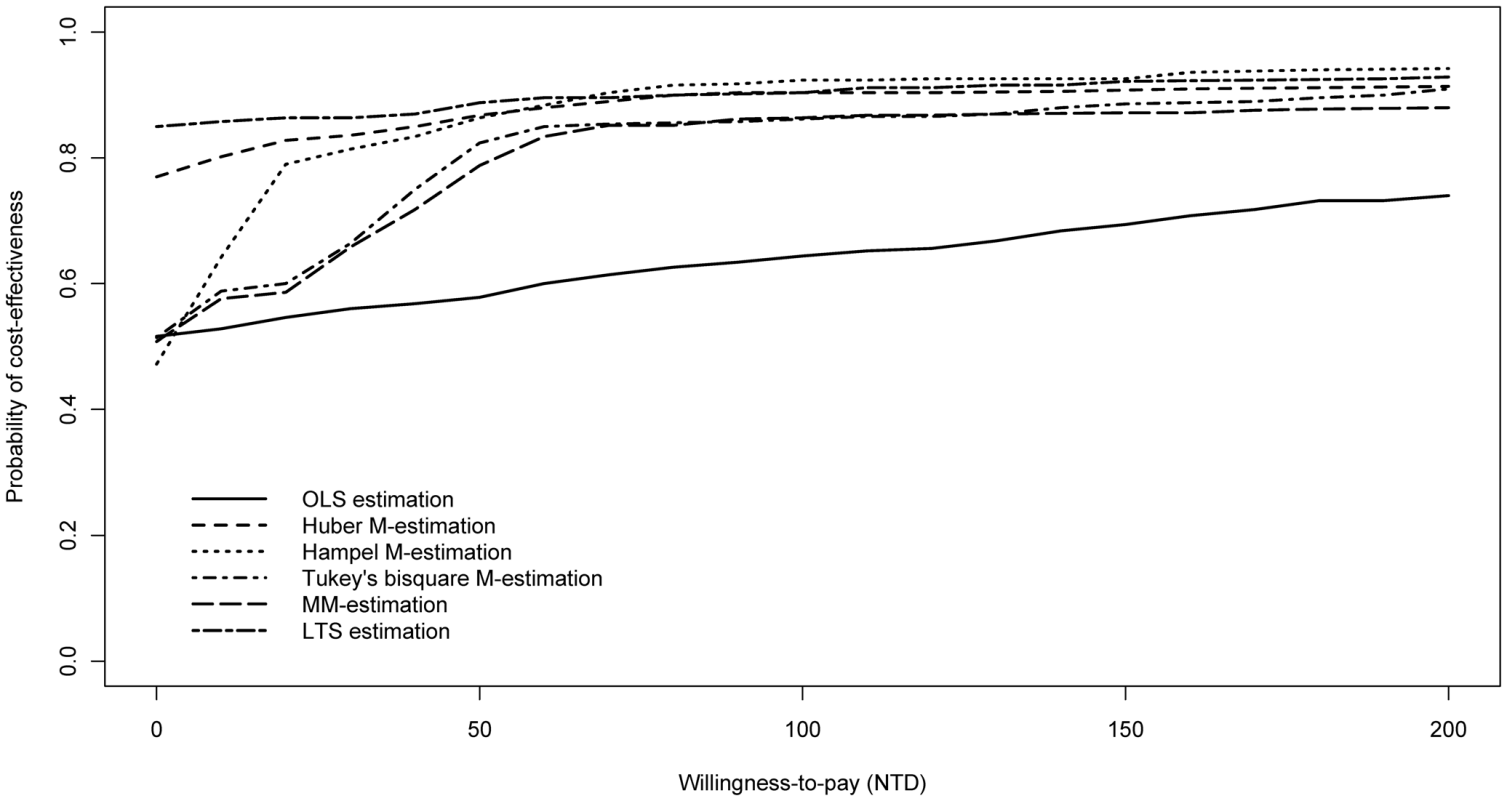
Cost-effectiveness acceptability curve based on different estimation procedures.

Table 4 showed the estimates of NBR using four values of 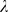 = 50, 100, 150 and 200. The proportions of outliers were around 4% to 16% given different values of WTP. The probability of cost-effectiveness (aspirin plus PPIs vs. clopidogrel) was calculated by regression and bootstrapping approach given different 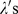. The estimated values of incremental net-benefit (regression coefficient on the treatment indictor) by OLS estimation were the lowest than those by robust estimations. The results generated by OLS have the lowest probabilities of being cost-effective (both based on regression and bootstrapping). All probabilities of cost-effectiveness of OLS estimation were under 0.75, much lower than the probabilities of other five robust regressions. Figure 1 shows the CEAC's estimated by different estimation procedures. Except for extremely low WTP, the CEAC by OLS estimation was below other CEACs by robust approaches. If the probability of cost-effectiveness is set to be 0.8, the critical value for aspirin plus PPIs being cost-effective compared to clopidogrel is about 200 NTD by robust methods and would be higher than 200 NTD by OLS estimation.

**Table 4:** Results of net benefit estimates and the probability of cost-effectiveness for six estimation procedures.

Estimation	WTP	Incremental net-benefit		Probability of cost-effectiveness (regression)	Probability of cost-effectiveness (bootstrapping)	Proportion of outliers (%)
		Estimate	One-sided p-value			
OLS estimation	50	1544.63	0.4663	0.53	0.58	-
Huber *M*-estimation		3523.60	0.1333	0.87	0.87	10
Hampel *M*-estimation		3422.04	0.1381	0.86	0.86	8
Tukey's bisquare *M*-estimation		2331.04	0.1662	0.83	0.82	16
*MM*-estimation		2010.86	0.1902	0.81	0.79	16
LTS-estimation		2505.48	0.1174	0.88	0.89	9
OLS estimation	100	3050.85	0.4341	0.57	0.64	-
Huber *M*-estimation		5211.37	0.1204	0.88	0.90	8
Hampel *M*-estimation		5853.44	0.0967	0.90	0.92	6
Tukey's bisquare *M*-estimation		3890.12	0.1582	0.84	0.86	10
*MM*-estimation		3957.80	0.1501	0.85	0.86	11
LTS-estimation		3484.71	0.1385	0.86	0.90	13
OLS estimation	150	4557.07	0.4029	0.60	0.69	-
Huber *M*-estimation		6659.34	0.1206	0.88	0.91	7
Hampel *M*-estimation		8415.03	0.0781	0.92	0.93	5
Tukey's bisquare *M*-estimation		5642.65	0.1397	0.86	0.89	8
*MM*-estimation		5416.11	0.1465	0.85	0.87	9
LTS-estimation		5726.75	0.1024	0.90	0.92	10
OLS estimation	200	6063.29	0.3733	0.63	0.74	-
Huber *M*-estimation		8093.44	0.1216	0.88	0.91	6
Hampel *M*-estimation		10485.46	0.0776	0.92	0.94	4
Tukey's bisquare *M*-estimation		7631.85	0.1234	0.88	0.91	7
*MM*-estimation		7102.43	0.1387	0.86	0.88	8
LTS-estimation		7355.56	0.0970	0.90	0.93	9

## Discussion

In this study, we presented simulations on different parameters to demonstrate the influence of outliers in estimating NBR for CEA. It was shown that the presence of outliers in cost data can lead to lower empirical powers under various outlier scenarios and higher empirical sizes for some scenarios in 20% and 30% of outliers, hence leading to incorrect decision making.

There were two important features in the simulation. The first feature was the consideration of outlier mechanism. Large outliers in cost variable were assumed to occur randomly. In practice, outliers can be caused by many reasons such as measurement error or data entry mistakes. Under such circumstance, the errors or mistakes could be corrected ad hoc and prevented beforehand. However, when outliers were not man-made and cannot be excluded from the analysis directly, CEA should be conducted with caution on some specific patient populations. In this context, cost or effect outliers are not attributed to the illness or treatment of interest. Instead, they occur primarily because of patients' complicated conditions, other severe medical history or old age which could incur higher medical costs. In such case, the reasons causing outlier become the confounding factors and direct deletion of outliers from the data may bias CEA results. To circumvent this, subgroup (subpopulation) analysis may be an alternative to such situation if one can distinguish between outliers and usual case [3]. However, it is often the case that outliers and usual cases are not directly distinguishable in empirical studies. In view of this, robust estimation provides a procedure to avoid the possible influence of outliers.

The second feature was that the scenario of hypothesis testing on cost-effectiveness was considered. When true net-benefit is non-positive, OLS and robust estimations performed almost equally well where chances of making wrong decision (type I error) were less than the statistical significance level 0.05 except when higher proportion of outliers is in the sample. However, the focus of this study was to point out the better performance of robust estimations over OLS one in terms of empirical power, i.e. declaring positive net-benefit while true net-benefit was positive. Specifically, it is worthwhile noting that when the proportion of outliers in the data is large, i.e. over 30%, general *M*-estimations such as Huber *M*-estimation, Hampel *M*-estimation and Tukey's bisquare *M*-estimation performed equally well as OLS estimation. LTS estimation and *MM*-estimation still produced robust results with high likelihood of making correct decisions, remaining uninfluenced by the proportion of outliers because of high breakdown point.

In the empirical example of antiplatelet therapy, robust estimations led to higher probability of claiming aspirin plus PPIs as cost-effective than clopidogrel given a set of WTPs. In Figure 1, CEAC of OLS estimation was well below those of robust estimations. As the WTP value increased, the CEAC of OLS estimation only slightly increased from 50% to around 60% while the five robust estimations attained above 80%. Compared to robust estimations, using OLS estimation would require a comparatively larger critical value to conclude that aspirin plus PPIs is cost-effective. This indicated that aspirin plus PPIs was considered more significantly cost-effective than clopidogrel in robust estimations while not in OLS estimation given an appropriate WTP.

One concern using robust estimation for net-benefit data is the issue of sample size. In the simulation, it was shown that the empirical power of all robust estimations were enhanced as sample size increased. Therefore, relatively larger sample size was required to ensure the reliability of CEA results in NBR. In summary, sample size, outlier distribution and proportion all played a major role in testing cost-effectiveness in NBR. Smaller sample size, serious departure of outlier distribution from target population and large outlier proportion would lead to erroneous results. Either increasing sample size or using robust approaches would reduce the impact of outliers. However, if the proportion of the outliers was over 20%, the performance of three types of *M*-estimation was almost equivalent or sometimes worse to that of OLS estimation. *MM*-estimation was especially suitable to deal with the outliers derived from extreme distribution and LTS estimation was almost dominant over other estimation in our simulated results. Cautious measures are strongly suggested when handling the case with small sample size, large proportion of outliers and extreme outliers.

In a nutshell, five robust estimations outperformed OLS estimation on hypothesis tests of cost-effectiveness. Among those robust estimations, LTS estimation provided a better result in testing cost-effectiveness and a higher probability of claiming cost-effectiveness of an intervention when it is actually cost-effective given a WTP. Tukey's bisquare *M*-estimation and *MM*-estimation performed almost as well as LTS estimation when the proportion of outliers was less than 30%. For more extreme outliers, *MM*-estimation performed equally well with LTS estimation. In summary, LTS estimation is recommended in practice when a NBR is applied for CEA.
